# Thrombospondin-1 in Urological Cancer: Pathological Role, Clinical Significance, and Therapeutic Prospects

**DOI:** 10.3390/ijms140612249

**Published:** 2013-06-07

**Authors:** Yasuyoshi Miyata, Hideki Sakai

**Affiliations:** Department of Nephro-Urology, Nagasaki University Graduate School of Biomedical Sciences, 1-7-1 Sakamoto, Nagasaki 852-8501, Japan; E-Mail: hsakai@nagasaki-u.ac.jp

**Keywords:** thrombospondin, urological cancer, TSP-1-derived peptide, therapy

## Abstract

Angiogenesis is an important process for tumor growth and progression of various solid tumors including urological cancers. Thrombospondins (TSPs), especially TSP-1, are representative “anti”-angiogenic molecules and many studies have clarified their pathological role and clinical significance *in vivo* and *in vitro*. In fact, TSP-1 expression is associated with clinicopathological features and prognosis in many types of cancers. However, TSP-1 is a multi-functional protein and its biological activities vary according to the specific tumor environments. Consequently, there is no general agreement on its cancer-related function in urological cancers, and detailed information regarding regulative mechanisms is essential for a better understanding of its therapeutic effects and prognostic values. Various “suppressor genes” and “oncogenes” are known to be regulators and TSP-1-related factors under physiological and pathological conditions. In addition, various types of fragments derived from TSP-1 exist in a given tissue microenvironment and TSP-1 derived-peptides have specific activities. However, a detailed pathological function in human cancer tissues is not still understood. This review will focus on the pathological roles and clinical significance of TSP-1 in urological cancers, including prostate cancer, renal cell carcinoma, and urothelial cancer. In addition, special attention is paid to TSP-1-derived peptide and TSP-1-based therapy for malignancies.

## 1. Introduction

Angiogenesis is a key process for tumor growth and cancer cell dissemination for nearly all solid malignant tumors. It is a multi-step process that involves differentiation, proliferation, migration, and tube-formation of endothelial cells and changes in the extracellular matrix under pathological conditions. Various cells, such as cancer cells, stromal cells, and tumor-associated infiltrating cells, participate in these regulative processes, and tumor-associated angiogenesis depends on the local balance between pro-angiogenic factors and anti-angiogenic factors in a given tissue microenvironment [1].

Various molecules, including vascular endothelial growth factor (VEGF), platelet-derived growth factor, and transforming growth factor (TGF), can induce angiogenesis [2]. The pathological roles and clinical significance of these “pro-angiogenic” molecules have been extensively studied *in vivo* and *in vitro*. Thus, these investigations have led to the development of agents with anti-angiogenic properties as treatments for various malignancies, including urological cancers [3,4].

The pathological functions and clinical significance of these anti-angiogenic molecules remain incompletely characterized. The purpose of the present review was to discuss experimental data regarding the class of potent anti-angiogenic factors, thrombospondins (TSPs), and their use for the treatment of urological cancers. Special focus is given to TSP-1, as it is the most well known and is a potent therapeutic target in various malignancies [5,6].

## 2. Structure and Function of Thrombospondin

**Figure 1:**
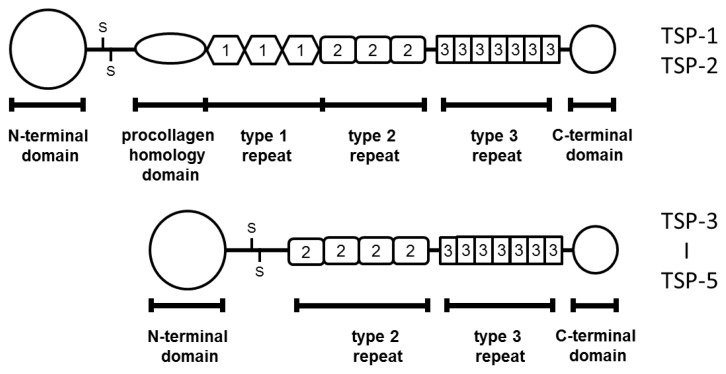
Structures of TSP members. Only TSP-1 and -2 have the type I repeat domain.

TSPs belong to a family of multi-domain and multi-functional calcium-binding extracellular glycoproteins. This family consists of five genes encoding proteins: TSP-1 to -5 [7]. They can be divided into two subgroups according to oligomerization state and domain structure [8]: trimeric proteins (TSP-1 and -2) and pentameric proteins (TSP-3 to -5) (Figure 1).

One important characteristic of the trimeric sub-group is the presence of a type 1 repeat domain that has a specific angiogenesis-related function [7]. Among these members, TSP-1 was the first family member to be identified. It was isolated from platelet α-granules in 1978 and is a specific product of platelet activation [9,10]. Various studies have demonstrated that TSP-1 regulates cell differentiation, proliferation, migration, and apoptosis of fibroblasts, smooth muscle cells, and macrophages [7,11–13]. Thus, TSP-1 plays an important role in the regulation of various biological activities, including vascular homeostasis, immunity, and wound healing [14–16].

TSP-2 is also expressed in various tissues and correlates with tissues remodeling and inflammation under physiological and pathological conditions [17,18]. As mentioned above, TSP-2 has a type 1 repeat domain, therefore, TSP-2 is speculated to play a role in the regulation of angiogenesis. However, detailed activities and pathological significance of TSP-2 are not fully understood. The biological activities and expression of TSP-1 are regulated by a wider variety of factors (including cytokines and growth factors) when compared with TSP-2 [17], and, TSP-1 is thought to play more important roles in the malignant phenotype when compared with TSP-2. By contrast, almost no information is available regarding the localization, expression, biological role, or pathological significance of TSP-3 to -5 in human tissues, particularly in the context of urological cancers. Thus, the present review primarily focuses on the pathological and clinical significance of TSP-1 in urological cancers.

### 2.1. TSP-1

TSP-1 is a well-studied glycoprotein that has four motifs (*i.e.*, adhesive domains; *N*-terminal domain, type I repeats, type III repeat) and a *C*-terminal domain [19]. TSP-1 suppresses endothelial cell proliferation, migration, and tube-formation and induces endothelial apoptosis [20–22]. Thus, TSP-1 is an endogenous inhibitor of angiogenesis under physiological and pathological conditions, including in the context of malignancy [7,23–25]. However, TSP-1 can also stimulate angiogenesis [26]. Furthermore, the angiogenesis-related function of TSP-1 is very complex; for example, it inhibited tube-formation of endothelial cell at 15 μg/mL, but enhanced tube-formation at 1–10 μg/mL [27]. Furthermore, the biological function and regulative mechanism of TSP-1 in endothelial cells are different when comparing the immobilized and soluble form [28,29]. In addition to such direct function for endothelial cells, TSP-1 also modulates the extracellular matrix and leads to degradation and remodeling of connective tissues [30], which can subsequently regulate angiogenesis. Furthermore, TSP-1 controls tissue perfusion and hemostasis by regulation of nitric oxide (NO) signaling [31]. Thus, the roles of TSP-1 in the regulation of angiogenesis are extremely complex and involve direct and indirect effects on stromal cells and the extracellular matrix. Regulation of angiogenesis and degradation of the extracellular matrix are also crucial steps for tumor growth, cancer cell invasion, and metastasis in nearly all solid tumors, and TSP-1 plays important roles for cancer cell invasion and dissemination in malignancies. In fact, the regulation of tumor growth and progression by TSP-1 has been described [24].

### 2.2. TSP-1 in Malignancies

TSP-1 had been recognized as a “tumor-suppressor” protein, based on the fact that it has anti-angiogenic function in various malignancies. However, numerous investigators also support the opinion that TSP-1 is a multi-functional protein and that its biological activities and pathological roles in malignancy are complex and controversial [32]. For example, although TSP-1 expression was increased in breast cancer and colorectal cancer [33,34], the opposite result was reported in esophageal cancer and glioblastoma [35,36]. In addition, conflicting results regarding the pathological significance and the role of TSP-1 in progression of malignant tumors have been described in animal experiments, human pathological studies, and comprehensive reviews [31,37]. Therefore, we will discuss the pathological roles of TSP-1 in malignant tumors separately in regards to angiogenesis, proliferation, and invasion.

Among published studies, there are conflicting results regarding the relationship between TSP-1 and angiogenesis in cancer patients. Although TSP-1 acts as an anti-angiogenic factor in various malignancies, including melanoma and cervical cancer [38,39], several reports have shown that TSP-1 levels were positively associated with microvessel density (MVD) in breast cancer [40] and gastric cancer [41]. By contrast, other reports demonstrated that TSP-1 expression did not correlate with MVD in ovarian cancer [26] or in cholangiocarcinoma [42]. Further, there are also discrepancies regarding the role of TSP-1 in cancer cell proliferation and invasion. With respect to tumor growth, several investigators have suggested that TSP-1 might directly suppress proliferation of cancer cells [43,44]. However, another study reported that TSP-1 expression did not affect cell proliferation in breast cancer [45]. In terms of cancer cell invasion, although TSP-1 expression was inversely correlated with cell invasion in thyroid cancer in one study [46], other studies reported that TSP-1 promotes cell invasion in breast cancer [47] and thyroid cancer [48]. In addition, in an animal model of breast cancer, knock-out of TSP-1 led to growth of the primary tumor but a decrease in the number of metastases [49]. Thus, the pathological roles and clinical significance of TSP-1 are complex, and there remains considerable disagreement on this matter. Finally, there are several reports that have demonstrated that increased TSP-1 expression was a poor prognostic marker for survival in several cancers including colorectal cancer [50,51]. By contrast, low levels of TSP-1 correlated with poor prognosis in colon cancer [52] and non-small cell lung cancer [53].

The localization of TSP-1 in cancer tissues may also be of significance. For example, altered TSP-1 expression has been detected in cancer cells; however, pathological and significant roles were found in increased TSP-1 originated from cancer cells, but not stromal cells [54,55]. Furthermore, induction of TSP-1 expression appears to be a paracrine effect of growth factors released by tumor cells and such paracrine induction was reported to play important roles in malignant aggressiveness in transgenic mice [56]. Thus, the significance of TSP-1 seems to vary, is possibly cell type- or tissue type-specific, and may depend on its localization with tumor tissues.

## 3. TSP-1 in Prostate Cancer

TSP-1 plays important roles in homeostasis, including regulation of angiogenesis in prostate tissues [57,58]. Since the 1990s, the angiogenic function and pathological roles of TSP-1 in prostate cancer have been investigated using various methodologies. At present, many investigators believe that TSP-1 is one of the most significant anti-angiogenic factors in prostate cancer [43,59,60]. However, other investigators suggest that TSP-1 is not always associated with angiogenesis in prostate cancer tissues [61,62]. On the other hand, TSP-1 has been reported to correlate with prostate cancer cell migration [51]. Furthermore, TSP-1 expression and function can change according to androgen-dependency [51,60]. Thus, TSP-1 may affect the various pathological characteristics and status of prostate cancer patients. The following section examines the pathological role, clinical significance, and predictive value of TSP-1 in prostate cancer.

### 3.1. TSP-1 Expression

Studies of TSP-1 expression in prostate cancer and non-tumoral prostate tissues have shown conflicting results, even when similar methods were used. For example, several studies have reported that TSP-1 is strongly expressed in normal tissues and, to a lower extent, in prostate cancer tissues [57,63]. In addition, Vallbo *et al.* [64] reported that TSP-1 expression was detected in benign prostatic hyperplasia (BPH) and prostatic intraepithelial neoplasia (PIN), whereas it was absent in all prostate cancer tissues (0/34). In regard to TSP-1 expression in cancer cell lines, one report demonstrated that TSP-1 expression was lower in DU145 and LNCaP than in SV-40-immortalized prostatic epithelial cells [65]. These findings support the hypothesis that TSP-1 acts as tumor-suppressor in prostate cancer. On the other hand, another study reported that TSP-1 expression was higher in prostate cancer cells than in benign tissues [51,66]. These results show that TSP-1 may act as a promoter of prostate cancer. Thus, the expression and carcinogenic activities of TSP-1 remain controversial.

### 3.2. Correlation with Clinicopathological Features

TSP-1 negatively correlates with prostate cancer cell proliferation *in vitro* [65]. Similarly, several other *in vivo* studies reported that TSP-1 inhibited tumor growth [60,67]. However, one study reported that TSP-1 had no significant influence of the proliferation and tumor growth of prostate cancer [68]. A similar discrepancy in the relationship between TSP-1 expression and angiogenesis has been described for human prostate cancer tissues. For example, TSP-1 expression was not associated with MVD in prostate cancer patients [61,66], while other studies reported that decreased TSP-1 levels correlated with an increase in MVD [51]. Another study reported that TSP-1 was positively associated with MVD [69].

The relationship between TSP-1 expression and clinicopathological characteristics, outcomes, and survivals are summarized in Table 1.

Contrary to expectation, more than half of the previous reports showed that TSP-1 status was not significantly associated with clinicopathological features and prognosis. Furthermore, TSP-1 expression was significantly lower in metastatic prostate cancer than in localized prostate cancer [63]. One study suggested that the pathological significance of TSP-1 mRNA levels in tumor tissues was different from that in peri-tumoral tissues [51]. Briefly, TSP-1 level was not associated with pT stage and frequency of relapse in peri-tumoral tissues but was associated with these parameters in tumoral tissues. Therefore, there is no general agreement regarding the pathological roles and clinical significance of TSP-1 expression in patients with prostate cancer.

### 3.3. TSP-1 and Androgen Therapy

Androgens stimulate and maintain prostate growth during development, and androgen withdrawal is an effective strategy for the treatment of prostate cancer. Studies have investigated the relationship between TSP-1 expression and hormonal therapy, androgen deprivation status, and hormone-sensitivity in prostate cancer, because the TSP-1 promoter contains a hormone response element that is sensitive to testosterone agonists [62,67]. An *in vitro* study using cancer cell lines showed that mRNA and protein levels of TSP-1 were very low in androgen-dependent LNCaP, although they were clearly detected in androgen-independent PC3 cell line [51]. In a rat model, castration led to an increase in TSP-1 expression and an inhibition of angiogenesis in prostate tissues [62]. Similar findings were also reported in a mouse model [58]. In addition, prostate cancer tissues obtained from patients undergoing androgen deprivation therapy showed significantly higher expression of TSP-1 when compared with those in patients who were not undergoing such therapy [58,63]. Furthermore, one study reported that TSP-1 expression is increased in human hormone-refractory tumor tissues [62] and that TSP-1 expression was no longer associated with a reduced MVD in castration-resistant prostate cancer (CRPC), despite the finding of a significant inverse correlation in androgen-dependent prostate cancer [62]. However, another study showed that TSP-1 is a potent anti-angiogenic factor and a trigger of cancer cell migration, but not of cell proliferation or apoptosis in CRPC [51]. In addition, when a TSP-1 expression vector was transduced into androgen-independent DU-145 cells, tumor growth was inhibited in a xenograft model [60]. On the other hand, another study reported that TSP-1 was detected at the same level in DU145, PC-3, and LNCaP cell-derived proteasomes [70]. Thus, the detailed function of TSP-1 in CRPC remains poorly understood.

## 4. TSP-1 in Renal Cell Carcinoma

Anti-angiogenic agents are useful for the management of advanced and recurrent renal cell carcinoma (RCC). Some of the main targets of these molecularly targeted therapies are the VEGFs, VEGF-receptors (Rs), and mTOR. Numerous studies of the pathological roles and clinical significance of these “pro”-angiogenic factors have been performed *in vivo* and *in vitro*. By contrast, there are fewer studies of “anti”-angiogenic factors when compared with “pro”-angiogenic factors. In this manuscript, we showed pathological roles and clinical significance of TSP-1 as a representative of “anti”-angiogenic factors. We also emphasize that more detailed and comprehensive studies of molecular biology can lead to a breakthrough in treatment of RCC.

### 4.1. Pathological Significance of TSP-1

Increased angiogenesis is an important representative characteristic of RCC; however, the pathological significance of TSP-1 in RCC is not fully understood. In fact, there are relatively few reports regarding TSP-1 expression in human RCC tissues (Table 2).

Further, there are conflicting findings among studies in terms of the prognostic role of TSP-1. For example, although we found that TSP-1 expression had no impact on survival in RCC [71], another study reported that TSP-1 expression was negatively associated with cause-specific survival in a multivariate analysis model including pathological features [74]. The independent predictive value of TSP-1 expression for survival was also confirmed in other multivariate analysis models [75]. However, another study reported that TSP-1 immunoreactivity was not associated with multifocality in 38 RCC patients [76]. In addition, TSP-1 plasma levels in the renal vein were similar to those in general venous blood, and there was no significant change in TSP-1 plasma levels before and after nephrectomy, despite the fact that VEGF levels did change [72]. Thus, more detailed and larger studies are necessary to further explore these issues.

In an *in vitro* study, the levels of TSP-1 mRNA and protein were similar when comparing RCC cell lines and renal epithelial cells (HNK) [73]. Another study described TSP-1 expression in clear cell RCC cell lines; however, the level of expression was not compared with that in normal renal cells [77]. Thus, unfortunately, there is little information regarding TSP-1 expression in RCC cell lines. On the other hand, an *in vitro* study showed that HNK cells secreted high levels of TSP-1, which rendered them non-angiogenic, whereas cancer cells secreted little TSP-1 and were angiogenic [73]. In addition, TSP-1 can regulate pathological functions including invasion, in an autocrine manner [77]. These observations suggest that both the expression level and secretion level of TSP-1 might be important in RCC. Several studies have showed TSP-1 expression in stromal tissues [71,74,76], but no reports have shown that TSP-1 expression in the cancer cell cytoplasm. Although such discrepancies might be related to difference in methodologies and patient background between studies, it is certain that more detailed studies are necessary to arrive at a definitive conclusion.

### 4.2. Correlation between TSP-1 Expression and Malignant Aggressiveness

One study reported that TSP-1 might have a direct effect on the proliferation of cancer cells [74]. However, another report showed that TSP-1 expression did not correlate with cancer cell proliferation [71]. On the other hand, overexpression of TSP-1 was reported to inhibit tumor growth of liver metastases but not lung metastases in an animal model [78]. These observations support the notion that the regulation of TSP-1 activity varies according to the specific tissue microenvironment. Indeed, angiogenesis-related function is most representative activity for TSP-1. However, while one study reported that TSP-1 expression was significantly and negatively associated with MVD [74], other reports showed no such relationship [71,76]. Another study reported that TSP-1 produced by clear cell RCC cell lines inhibited cell migration in response to various chemoattractants [77].

## 5. TSP-1 and Urothelial Cancer

One of the most important characteristics of urothelial cancer (UC) is its high frequency of recurrence after initial treatment, even if only non-muscle invasive disease is present. In addition, approximately one quarter of these patients progress to muscle invasive disease [79]. Furthermore, patients with muscle-invasive cancer cells are at high risk for metastasis, and they often have poor prognosis due to the presence of disseminated cancer cells. These invasion- and metastasis-related steps are regulated by complex mechanisms, including tumor growth, degradation of stromal tissues, cell migration, and angiogenesis. Therefore, detailed information regarding these mechanisms is essential to formulate appropriate treatment and observation strategies for UC patients.

### 5.1. Pathological Significance

In regard to angiogenic function of TSP-1 in urothelial cancer, several reports showed TSP-1 expression was negatively associated with MVD [80,81]. In contrast to these reports, Ioachim *et al.* [82] reported a positive correlation between TSP-1 expression and MVD. Such a discrepancy was explained by the difference of methodology including the specific antibody, condition of specimens, and method of evaluation.

In a study of cancer cell lines, TSP-1 was expressed in less aggressive cell lines (MGH-U4 and RT-4) but not in the more aggressive cell lines (RT-112 and UMUC-3) [83]. In addition, TSP-1 expression in bladder cancer tissues was lower than that in normal bladder tissues [84]. These findings support the notion that TSP-1 has tumor-“suppressive” function in bladder cancer. On the other hand, various agents and biological substances, including valproic acid and androgens decreased TSP-1 levels in an animal model of bladder cancer [84,85]. Thus, TSP-1 expression and activity might be regulated by complex mechanisms. In fact, several studies showed that lower levels of TSP-1 expression correlated with malignant aggressiveness, including poor differentiation [82], tumor progression [86], and prognosis [80]. Similar findings have also been reported in patients with non-muscle-invasive bladder cancer [81]. Those studies suggested that TSP-1 was a tumor-suppressor of UC. However, different studies have reported conflicting findings regarding the relationship between TSP-1 expression and clinicopathological features, progression, and outcome in UC patients. For example, one study reported that TSP-1 immunostaining of cancer cells was negatively associated with grade and pT stage, but not with lymph node metastasis [86], while another study reported that TSP-1 expression was significantly associated with pathological stage and lymph node metastasis [87]. Further, Grossfeld *et al.* [80] reported that TSP-1 expression was not associated with pathological features, including pathological grade, stage, and the presence of lymph node metastasis.

### 5.2. Correlation between TSP-1 Expression and Prognosis

Shariat *et al.* [87] reported that downregulation of TSP-1 expression was an independent predictor of recurrence-free and cause-specific survival within a multivariate analysis model. In addition, other investigators also showed that altered TSP-1 expression was independently associated with recurrence-free and overall mortality in patients undergoing radical cystectomy [80]. However, multivariate analyses in that study also showed that a similarly significant result was found in recurrence-free survival for organ-confined tumors and in overall survival for lymph node metastasis-positive tumors. By contrast, this relationship was not present in regard to recurrence of tumors with extra-vesical extension, in tumors with lymph node metastasis, or in overall survival for organ-confined or extravesical extension. In an animal model of skin cancer, TSP-1 was negatively associated with angiogenesis and distant metastasis, whereas it was not associated with lymphangiogenesis or lymph node metastasis [56].

In bladder cancer, the pathological significance of TSP-1 expression in cancer cells was different from that in stromal cells. Briefly, tumor grade was negatively associated with tumor cell TSP-1 expression but not with stromal TSP-1 expression [82]. In contrast, although stromal TSP-1 expression in larger tumors as higher than that in smaller tumors, a similar relationship was not detected in terms of TSP-1 expression on tumor cell. In addition, this study also showed that TSP-1 expression in muscle invasive bladder cancer (MIBC) (pT2–4) was higher than that in pT1 tumors in terms of both tumor and stromal TSP-1. Furthermore, tumor and stromal TSP-1 expression was positively correlated with MVD. These data suggest that TSP-1 is an inducer of angiogenesis. Another study reported that the perivascular TSP-1 staining score was an independent predictor of disease progression within multivariate analysis [81]. By contrast, tumor cell and stromal TSP-1 expression were not independent predictors of disease progression according to univariate analysis in the same samples. These results are summarized in Table 3.

## 6. Molecular Regulation by TSP-1

TSP-1 is a multi-functional protein that exerts direct and indirect effects according to specific pathological conditions and organs. Many molecules are influenced by TSP-1 in malignancies. One of the mechanisms by which TSP-1 influences tumor invasion and cancer cell dissemination is through the regulation of a variety of proteolytic enzyme families, including urokinase-type of plasminogen activator (uPA) and matrix metalloproteinase (MMP) [48,88,89]. Among MMPs, Qian *et al.* [27] reported that TSP-1 is capable of stimulating MMP-9 production in bovine aortic endothelial cells. In malignancies, TSP-1 suppressed MMP-9 activation in breast cancer [43], and TSP-1 expression negatively and significantly correlated with MMP-9 expression in human UC tissues [86]. However, further study is needed because this negative correlation of UC had weak statistical power (*r* = −0.21). MMP-2 is another well-known molecule among the MMP family and plays an important role in tumor progression and survival in urological cancers. For example, TSP-1 inhibits MMP-2 activity by preventing activation of pro-MMP2 [89]. In addition, endocytic clearance of pro-MMP-2 is mediated by low density lipoprotein receptor-related protein (LRP) depending on TSP-1 [90]. On the other hand, TSP-1 signaling though LRP/calreticulin was positively associated with endothelial cell migration [91]. Furthermore, the pathological function of MMPs *in vivo* is regulated by a balance between MMPs and its physiological inhibitor, tissue inhibitor of metalloproteinases (TIMPs). Interestingly, TSP-1 can regulate TIMP-1 production in human tumor cells, including those derived from prostate cancer [92]. Thus, TSP-1 controls MMP signaling and MMP-related activities via complex mechanism.

Type I plasminogen inhibitor (PAI-1) is a serine protease that catalyzes the conversion of plasminogen to plasmin and that is a member of uPA family. PAI-1 is a well-known inhibitor of cell migration and angiogenesis; therefore, PAI-1 might inhibit cancer cell invasion and progression in malignant tumors, including prostate cancer and RCC [93,94]. However, one study reported that PAI-1 expression significantly and inversely correlated with TSP-1 expression in 162 RCC patients and that PAI-1 expression was promoter of tumor progression [75]. Thus, the biological and pathological regulation of PAI-1 expression in cancer tissues is complex and remains controversial.

The p53 tumor suppressor regulates angiogenesis though the modulation of TSP synthesis. In other words, wild-type p53 protein results in increased TSP-1 expression [95]. In fact, alterations of p53 are correlated with TSP-1 expression in colorectal cancer [96]. However, this relationship between p53 and TSP-1 was not detected in cholangiocarcinoma [97]. Among urological cancers, low TSP-1 expression was significantly associated with positive p53 status in RCC [74,98], bladder cancer [80], and prostate cancer [69], but several studies have reported that there was no significant correlation between TSP-1 expression and p53 status in prostate cancer [61,63] and urothelial cancer [82].

Hepatocyte growth factor (HGF) can affect cell biological behavior though downregulation of TSP-1 in ovarian cancer and thyroid cancer [99–101]. HGF downregulates TSP-1 via the (microtubule affinity-regulating kinase) MARK signaling pathway, leading to ovarian cancer cell invasion [101]. TSP-1 immunoreactivity was negatively associated with HGF immunoreactivity in human ovarian cancer tissues [100]. HGF and its pathway are important for tumor growth, prognosis, and survival in patients with urological cancers. Therefore, consideration of TSP-1-related HGF function is important when trying to formulate treatment strategies.

Expression and/or alterations of the *MYC* oncogene play important roles in many types of tumor cells including urological cancers [102,103]. For example, *MYC* expression in RCC tissues was significantly higher than that in normal tissues [103]. In addition, they also found that *MYC* expression was associated with cancer cell proliferation, anchorage-independent growth, and cell cycle in RCC cell lines. On the other hand, anomalies and alterations of c-Myc correlated with malignant potential and progression of prostate cancer [104] and bladder cancer [105]. Several animal studies have shown that c-Myc is a useful and potential therapeutic target for RCC [106,107]. Based on these facts, detailed information regarding the regulative mechanism and pathological roles of c-myc is important in order to facilitate selection of optimal treatment strategies for patients with malignancies, including urological cancers. Previous reports showed that c-Myc is a regulator of TSP-1 function under various pathological conditions [108–110]. Furthermore, a more recent report showed that TSP-1 signaling inhibits c-Myc expression in endothelial cells [111]. Therefore, further studies are necessary to characterize the relationship between c-Myc and TSP-1 in urological cancers.

Previous studies have shown that various tumor suppressor genes and oncogenes, such as *nm23*, *c-jun*, and *ras*, can regulate TSP-1 expression [111–113]. In addition, *cyclooxygenase (COX)-2*, *phosphatase*, *tensin analog* (*PTEN*) and *SMAD4* as tumor suppressors, or *myc* and *src* as oncogenes can repress TSP-1 production [114,115]. However, the regulatory mechanisms of TSP-1 expression in urological cancers are not fully understood. ELL-associated factor-1 (U19/EAF2) is a potential tumor suppressor that might regulate TSP-1 expression via blocking of p53-mediated repression of the TSP-1 promoter [116]. Interestingly, this molecule was identified based on its androgen responsiveness in the prostate [117], and its expression is downregulated in advanced prostate cancer tissues [118]. In addition, transfection of prostate cancer cell lines with U19/EAF2 resulted in apoptosis and decreased tumor growth in a xenograft tumor model [118]. On the other hand, hypoxia is an important regulator of TSP-1 in RCC [77]. Furthermore, although TSP-1 directly inhibits VEGF signaling, TSP-1 also inhibits VEGFR-2 signaling through engaging CD47 in endothelial cells [119]. Such blocking of interaction between CD47 and VEGFR-2 is most likely the domain manner by which TSP-1 inhibits VEGF activity. Thus, many factors are regulated by TSP-1 in various situations, such as tumor growth, progression, and angiogenesis.

## 7. Fragments Derived from TSP-1

**Figure 2:**
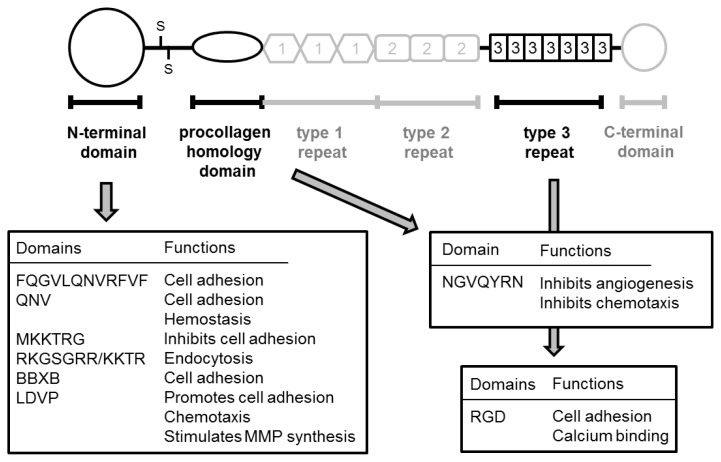
Pathological function of TSP-1-derived peptides in *N*-terminal, procollagen homology, and type 3 repeat domains.

**Figure 3:**
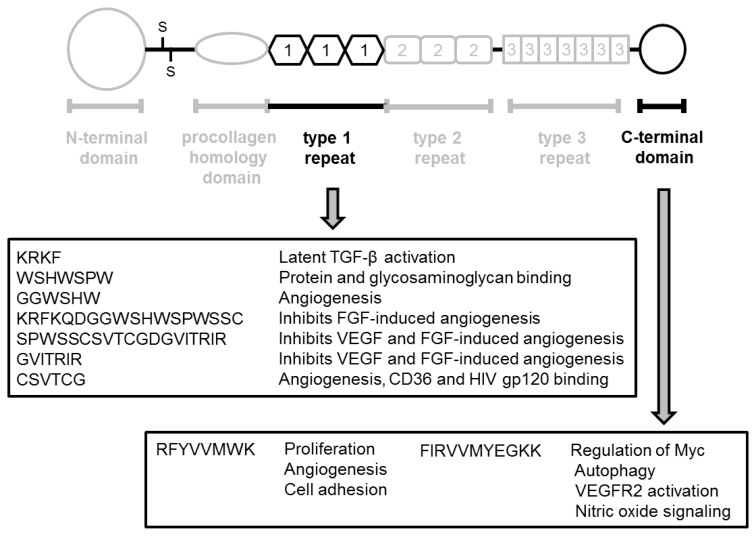
Pathological function of TSP-1-derived peptides in type 1 repeat and *C*-terminal domains.

TSP-1 plays various pathological roles in malignant tumors, especially in angiogenesis, cell proliferation, and invasion. In addition, the relationship between TSP-1 expression and clinicopathological features, progression, and prognosis seems to depend on the type of tumor. The specific methodology used including the antibody, are considered to account for these differences. On the other hand, we also speculated that a difference in fragments derived from TSP-1 in a given tissue microenvironment might account for the discrepancy in experimental results. For example, several TSP-1-derived peptides, such as *N*-terminal and type I repeats, have pro-apoptotic function in various cells, including malignant cells [120,121]. Further, NGVQYRN, called Col I overlap, derived from procollagen homology domain promotes survival of endothelial cells *in vivo* [122]. The CD47-binding peptide 7N3 (FIRVVMYEGKK) inhibits VEGF-stimulated cell impedance of endothelial cells [119]. In addition, the type 3 repeat/*C*-terminal domain regulates proliferation, differentiation, and cell death in malignant cells [123,124]. On the other hand, overexpression of the *N*-terminal fragment of TSP-1 resulted in less angiogenesis and an increase in invasiveness in a glioblastoma model [125]. TSP-1 was positively associated with TIMP-1 in a prostate cancer cell line (PC-3) [92]. In addition to these pathological activities, TSP-1 and TSP-1-derived peptide also plays important roles in apoptosis of cancer cells [126]. We showed specific functions of various peptides derived from TSP-1 in Figures 2 and 3.

Thus, specific function of TSP-1-derived peptides revealed by these studies is essential for an understanding of the biological, physiological, and pathological roles of TSP-1.

## 8. Treatment with TSP-1 Mimetic Drugs

The data described indicated that TSP-1 might be a therapeutic agent for the treatment of malignancies [5,126,127]. ABT-510 is a TSP-1-derived anti-angiogenic agent with promising candidate anti-tumoral agent, but phase II studies of this substance showed that it did not have anti-tumor effects in melanoma or sarcoma [128,129]. This drug was used for the treatment of prostate cancer in animal studies [130]. However, the combination therapy of ABT-510 and low-dose cyclophosphamide, cisplatin, and docetaxel has not been studied in patients with prostate cancer. Similarly, although ABT-510 was used for blocking the growth of bladder cancer in an animal model, this agent has not been studied in clinical trials of bladder cancer [131]. A phase II study of ABT-510 was performed for patients with advanced RCC [132]. Unfortunately, however, there was little evidence of clinical activity for ABT-510. Although treatment-related serious adverse events were relatively rare, vascular diseases including deep vein thrombosis occurred in these clinical trials [129,132].

Tasquinimod (ABR-215050) is also a TSP-1-derived anti-tumoral agent that results in improved progression-free survival (median = 7.6 months) when compared with placebo (3.3 months) in phase II clinical trial of patients with metastatic CRPC [6]. Based on this result, phase III trial are currently underway. Although tasquinimod is an orally active quinolone-3-carboxamide and although it had anti-tumor effects against prostate cancer growth in various experimental models and in a phase I study [133,134], the precise mechanism of its therapeutic effect is not fully understood. However, there is a general agreement that tasquinimod acts via inhibition of angiogenesis and that a part of its anti-angiogenic function is modulated by TSP-1 expression [133,135]. One study reported that tasquinimod increased TSP-1 levels and inhibited metastasis in a CRPC model [136]. In addition to the direct anti-tumoral effects of TSP-1-derived agents, an interaction between TSP-1 and tumor sensitivity to radiation have been recognized as one of several interesting treatment strategies of malignancies. For example, a recent study showed that suppression of CD47 led to a decrease in cell death in gastrointestinal tissues and an increase in peripheral circulating blood cell count in mice that were exposed to total body irradiation [137]. Another study reported that TSP-1/CD47 signaling was associated with radiation injury in normal tissues [138]. In addition, there is a report that inhibition of CD47 signaling resulted in an increase in the radio-sensitivity of tumors and radioprotection in normal tissues [139]. Thus, the combination of TSP-1-targeted agents and radiation may be a useful treatment strategy for cancer patients. Several studies have reported that TSP-1 may induce resistance against anti-cancer agents [114,140]. Interestingly, CD47-binding peptide (RFYVVMWK), which is derived from the *C*-terminal domain of TSP-1, prevents camptothecin- and doxorubicin-induced apoptosis in human thyroid carcinoma cells [140]. Thus, in addition to radiation therapy, the combination of TSP-1 mimetic agents and conventional chemotherapy are also expected to be effective.

Many reports have investigated CD47-mediated anti-tumoral activities in various malignancies. For example, one study reported that apoptosis of breast cancer cells was mediated by CD47-mediated pathways [126]. Furthermore, a recent study showed that TSP-1 signaling via CD47 inhibited c-Myc expression [141]. As discussed earlier, c-Myc plays important roles in malignant behavior and may be a therapeutic target in urological cancers. Similarly, the physiological and pathological roles of TSP-1 activation through CD47 signaling have been well-characterized. For example, TSP-1 signaling through CD47 plays as an inhibitor of several self-renewal transcription factors [141]. These observations suggest that CD47 is a therapeutic target for various malignancies. In fact, one study reported that a monoclonal antibody for CD47 exerted anti-tumoral activity via induction of cell death in leukemia cells [142]. Unfortunately, there were relatively few studies on other TSP-1 receptor-mediated activities, especially at the physiologic level. The CD36–TSP-1 pathway plays an important role in tumor angiogenesis and growth in several cancers including prostate and bladder cancer [51,143]. TSP-1 is exceedingly large and multifunctional. Therefore, comprehensive study of TSP-1-related interactions is needed to delineate the utility of TSP-1 mimetic drugs. Finally, as they are accompanied by further accumulation of new information and the development of TSP-1-derived peptides and TSP-1 mimetics, they hold great promise as future agents for the treatment of malignancies [37]. We also believe that combined therapies that incorporate TSP-1-derived peptide-targeted agents will be useful for the treatment of various malignancies, including urological cancer.

## 9. Conclusions

This review discussed the structure and biological activities of TSPs, especially TSP-1. The pathological roles and clinical significance of TSP-1 are dependent on the specific microenvironment and organ. In addition, TSP-1-derived peptide and domain should be noted for discussion regarding biological and pathological activities of TSP-1. Furthermore, this review discussed the regulatory mechanism of TSP-1 and TSP-1-related molecules. Finally, we described the relationship between TSP-1 and clinicopathological features, progression, and outcome in patients with urological cancers. TSP-1 was closely associated with many types of cancer-related factors. In addition, TSP-1 and some TSP-1 mimetic agents can enhance anti-tumor effects of a variety of chemotherapies and radiotherapies. These observations suggest that TSP-1 is a therapeutic target and prognostic factor, but further study is needed.

## Figures and Tables

**Table 1:** Correlation between TSP-1 expression and clinicopathological features in prostate cancer.

Year	*N*	Method	Change *	GS	pT stage	Metastasis	Prognosis	Ref.
2001	98	p-IHC	–	Neg.	–	–	Not S: survival	[69]
2002	85	p-IHC	–	Not S	–	–	Not S: survival	[61]
2002	82	p-IHC	Decrease	Not S	–	Neg.	–	[63]
2004	34	p-IHC	Decrease	–	–	–	–	[64]
2005	60	p-IHC	–	Neg.	–	–	–	[62]
2007	55	p-IHC	Increase	Not S	–	–	–	[66]
2011	35	RT-PCR **	–	Not S	Pos.	–	PSA relapse	[51]
2011	35	RT-PCR ***	–	Not S	Not S	–	Not S	[51]

GS: Gleason score; Ref: reference; p-: paraffin-embedded; IHC: immunohistochemistry; Not S: not significant; Neg.: negatively; Pos.: positively.

* Change of TSP-1 levels by carcinogenesis;

** m-RNA expression in tumoral tissue;

*** m-RNA expression in peri-tumoral tissue.

**Table 2:** Correlation between TSP-1 expression and clinicopathological features in renal cell carcinoma.

Year	*N*	Method	Change *	Grade	TNM stage	pT stage	Metastasis	Prognosis	Ref.
2003	119	p-IHC	–	–	–	Not S	Not S	–	[71]
2007	74	blood	–	Not S	Not S	–	–	Not S	[72]
2007	17	p-IHC	Not change	Neg.	–	–	–	–	[73]
2009	172	p-IHC	–	Neg.	Neg.	–	–	For survival	[74]

Ref: reference; p-: paraffin-embedded; IHC: immunohistochemistry; Not S: not significant; Neg.: negatively; Pos.: positively;

* Change of TSP-1 levels by carcinogenesis.

**Table 3:** Correlation between TSP-1 expression and clinicopathological features in patients with urothelial cancer.

Year	*N*	Method	Change *	Grade	Stage	pT stage	Metastasis	Prognosis	Ref.
1997	163	p-IHC	–	Not S	Not S	–	Not S	For survival	[80]
2002	220	p-IHC	–	Not S	Not S	–	–	For survival **	[81]
2006	148	p-IHC ***	–	Neg.	–	Neg.	–	–	[82]
2006	148	p-IHC ****	–	Not S	–	Neg.	–	–	[82]
2008	10	WB	Decrease						[84]
2009	131	p-IHC	–	Neg.	–	Neg.	Not S	–	[86]
2010	204	p-IHC	–	–	Neg.	–	Neg. for LN	For survival	[87]

Ref: reference; p-: paraffin-embedded; IHC: immunohistochemistry; Not S: not significant; Neg.: negatively; WB: Western blot; LN: lymph node;

* Change of TSP-1 levels by carcinogenesis;

** peri-vascular staining;

*** tumoral expression;

**** stromal expression.
